# Data for phase angle shift with frequency

**DOI:** 10.1016/j.dib.2016.04.008

**Published:** 2016-04-11

**Authors:** T. Paul, D. Banerjee, K. Kargupta

**Affiliations:** a Department of Physics, Indian Institute of Engineering Science and Technology, Shibpur, Howrah, India; b Department of Chemical Engineering, Jadavpur University, Jadavpur, Kolkata, India

**Keywords:** Phosphoric acid fuel cell, Impedance spectroscopy, Phase angle, Electrochemical reaction time

## Abstract

Phase angle shift between the current and voltage with frequency has been reported for a single phosphoric acid fuel cell in the cell temperature from 100 °C to 160 °C and the humidifier temperature from 40 °C to 90 °C. An electrochemical workbench is employed to find the shift. The figure of phase angle shift shows a peak in high humidifier temperatures. The peak in phase angle shift directs to lower frequency side with decreasing humidifier temperature. The estimation of electrochemical reaction time is also evaluated in the humidifier temperature zone from 50 °C to 90 °C.

## **Specifications Table**

**Table:** 

Subject area	*Physics*
More specific subject area	*Phosphoric Acid Fuel Cell, Electrochemistry*
Type of data	*Graph, Table*
How data was acquired	*An electrochemical workbench (AUTOLAB Model: PGSTAT 302N) was attached with a single phosphoric acid fuel cell. 4 probe electrochemical method was also applied to collect the data.*
Data format	*Raw and analyzed*
Experimental factors	*A wide range of frequency say 0.001 Hz to 10 kHz with sinusoidal excitation of* 10* mV was applied across the cell. Electrochemical impedance spectroscopy was used to find the data. The entire datum was taken at cell temperatures of 100–160 °C and humidifier temperatures of 40–90 °C. The experimental error in the phase angle measurement is within ±0.01*°*.*
Experimental features	*A single phosphoric acid fuel cell was constructed and an electrochemical workbench was connected using two probe configuration method.*
Data source location	*Jadavpur University, Kolkata, India*
Data accessibility	*Data is with this article*

## Value of the data

• Phase angle shift with frequency is commonly used yet powerful technique for electrochemical analysis.
• Other researchers can use the data as guidelines to select these parameters for their fabricated fuel cell without rigorous trials and errors.
• The designed technique can also be applicable for polymer electrolyte membrane fuel cells as well as solid oxide fuel cell.

## 1. Data

Shift in phase angle between the current and voltage is evaluated by applying sinusoidal excitation of 10 mV across the single phosphoric acid fuel cell. Figures depicting the variation of phase angle with frequency are shown here in wide cell temperatures and humidifier temperatures.

## 2. Experimental design, materials and methods

**Fig. 1:**
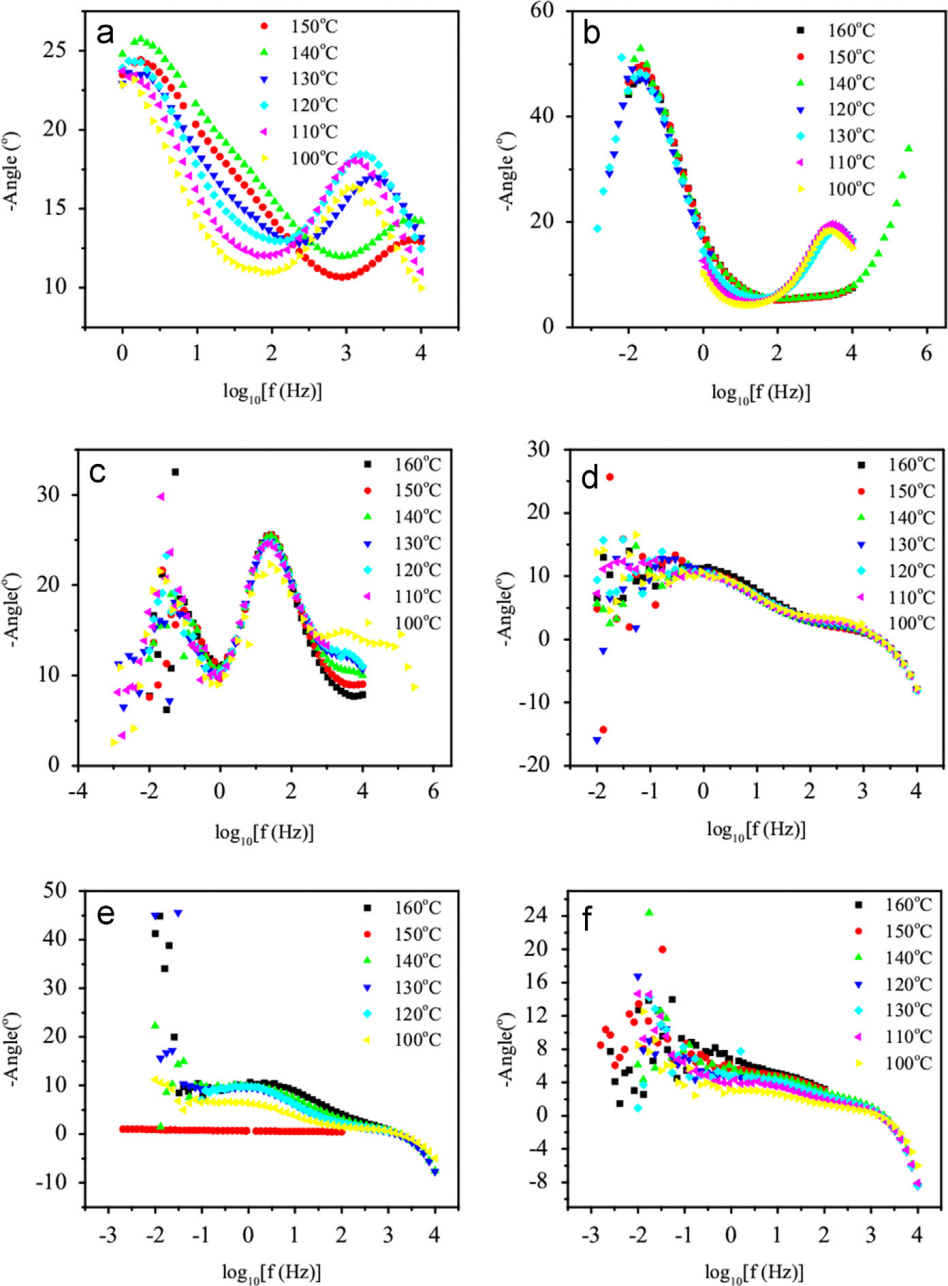
Frequency dependence of phase angle shift for single phosphoric acid fuel cell in wide cell temperatures and humidifier temperatures: (a) 90 °C (b)80 °C (c) 70 °C (d)60 °C (e) 50 °C and (f) 40 °C.

A single unit of phosphoric acid fuel cell (PAFC) consists of anode, electrolyte and cathode. Here, 88 wt% phosphoric acid (Merck, India) was used as an electrolyte. The details of the experimental set up were also reported elsewhere [1], [2]. A glass mat soaked in the phosphoric acid was used as a solid electrolyte. Two electrodes were composed of thin layers of 20 wt% Pt/C, as deposited on to carbon plate. The electrolyte/electrode assembly was placed between two grooved graphite plates. Pure H_2_ was passed through a humidifier to humidify the gas and fed to the anode through the grooved graphite plates. In the cathode, the O_2_ gas was fed. The outlets of the grooved graphite plates from both cathode and anode were connected to an adsorber to collect moisture. Two stainless steel plates were placed at the two ends and were used as current collectors. A heating plate was placed on the lower current collector to maintain the cell temperature. The total arrangement was kept intact by using two pusher plates. Throughout all the experiments the inflows of H_2_ and O_2_ gases were maintained at 100 and 50 cc/min, respectively using rotameters. Measurement of phase angle shift between the current and voltage using the electrochemical workbench was performed after 3 h of starting the gas flow. This time is given so that the open circuit potential was generated after the electrochemical reactions inside the cell. As parameters both the cell temperature and humidifier temperature were varied. The variation of phase angle shift with frequency is shown in Fig. 1. A well-defined peak in phase angle shift is observed for higher humidification [Fig. 1(a)–(e)]. The peak shifts towards lower frequency region as the humidifier temperature decreases in Fig. 1(a)–(e). It is observed from Fig. 1(f) that the peak vanishes at 40 °C humidifier temperature. Scattering of datum in extreme low frequency indicates the uncertainty of measurement beyond the experimental error [Fig. 1(d)–(f)]. The electrochemical reaction time (*τ*) is evaluated using the peak of phase angle shift at the investigated temperatures [3]. The electrochemical reaction time is also presented in Table 1. It is found that *τ* has maximum 0.8 ms and minimum 1.45 s for higher humidifier temperature and lower humidifier temperature respectively.

## Figures and Tables

**Table 1:** Estimation of electrochemical reaction time (*τ*) at different cell temperatures and humidifier temperatures.

Cell temperature (°C)	Humidifier temperature (°C)	Electrochemical reaction time (*s*)
150	90	1.20×10^−4^
140	90	1.45×10^−4^
130	90	4.11×10^−4^
120	90	5.43×10^−4^
110	90	6.79×10^−4^
100	90	8.39×10^−4^
120	80	3.09×10^−4^
110	80	3.72×10^−4^
100	80	4.49×10^−4^
160	70	0.037
150	70	0.037
140	70	0.037
130	70	0.037
120	70	0.049
110	70	0.039
100	70	0.039
160	60	0.82
150	60	0.62
140	60	0.82
130	60	1.09
120	60	0.82
100	60	1.09
160	50	0.44
140	50	0.47
130	50	1.21
120	50	1.04
100	50	1.45
